# Nutritional support in hospitalised patients with diabetes and risk for malnutrition: a secondary analysis of an investigator-initiated, Swiss, randomised controlled multicentre trial

**DOI:** 10.1136/bmjopen-2024-084754

**Published:** 2024-08-16

**Authors:** Bettina Keller, Carla Wunderle, Pascal Tribolet, Zeno Stanga, Nina Kaegi-Braun, Beat Mueller, Philipp Schuetz

**Affiliations:** 1 Kantonsspital Aarau AG, Aarau, Switzerland; 2 Faculty of Medicine, University of Bern, Bern, Bern, Switzerland; 3 Karolinska Institutet, Stockholm, Stockholm, Sweden; 4 Medical Faculty Department of Clinical Research, University of Basel, Basel, Basel-Stadt, Switzerland; 5 Medical University Department, Clinic for Endocrinology, Cantonal Hospital Aarau, Aarau, Aargau, Switzerland

**Keywords:** Nutritional support, NUTRITION & DIETETICS, Randomized Controlled Trial, DIABETES & ENDOCRINOLOGY

## Abstract

**Objectives:**

The main objective of this study was to investigate the effects of nutritional support on mortality in hospitalised patients with diabetes and nutritional risk participating in the *Effect of early nutritional support on Frailty, Functional Outcomes, and Recovery of malnourished medical inpatients Trial* (EFFORT) trial.

**Design:**

Secondary analysis of a Swiss-wide multicentre, randomised controlled trial.

**Participants:**

Patients with diabetes and risk for malnutrition.

**Interventions:**

Individualised nutritional support versus usual care.

**Primary outcome measure:**

30-day all-cause mortality.

**Results:**

Of the 2028 patients included in the original trial, 445 patients were diagnosed with diabetes and included in this analysis. In terms of efficacy of nutritional therapy, there was a 25% lower risk for mortality in patients with diabetes receiving nutritional support compared with controls (7% vs 10%, adjusted HR 0.75 (95% CI 0.39 to 1.43)), a finding that was not statistically significant but similar to the overall trial effects with no evidence of interaction (p=0.92). Regarding safety of nutritional therapy, there was no increase in diabetes-specific complications associated with nutritional support, particularly there was no increase in risk for hyperglycaemia (adjusted OR 0.97, 95% CI 0.56 to 1.67 p=0.90).

**Conclusion:**

Patients with diabetes and malnutrition in the hospital setting have a particularly high risk for adverse outcomes and mortality. Individualised nutritional support reduced mortality in this secondary analysis of a randomized trial, but this effect was not significant calling for further large-scale trials in this vhighly ulnerable patient population.

**Trial registration number:**

NCT02517476.

**STRENGTHS AND LIMITATIONS OF THIS STUDY**

- Analysis of the clinical effects of nutrition in a large sample of hospitalised patients with diabetes and nutritional risk.
- Prospective randomised controlled design with detailed clinical characterisation of patients.
- Long-term follow-up of patients regarding clinical endpoints.
- Limitations include the secondary analysis and the lack of diabetes-specific nutritional treatment and information in some patients including HbA1c levels and continuous glucose levels during the hospital stay, as well as the lack of a protocol among centres for the treatment of hyperglycaemia.

## Introduction

 The prevalence of type 2 diabetes has substantially increased worldwide,^1^ particularly among the elderly, polymorbid patient population with various chronic conditions, including chronic kidney or cardiovascular disease.^2^ Diabetes per se is a risk factor for worse clinical outcomes across a wide spectrum of medical conditions, such as infections,^3^,^6^ heart failure^7 8^ or cancer,^9 10^ and also represents a substantial economic burden.^11^ The risk for adverse events is further increased if patients with diabetes develop disease-related malnutrition,^12^ which affects between 60% and 80% of all hospitalised patients.^13 14^ The risk of malnutrition among patients with diabetes may be explained by several factors, including diabetes medication that affect appetite (eg, GLP-1 agonists),^13^ low-grade inflammation associated with diabetes, older age or dietary habits focusing on glycaemic and weight control rather than optimal nutritional intake among others.^15 16^ While the risk to develop malnutrition in hospitalised patients with diabetes is well established, there is a lack of data exploring the best therapeutic approach to prevent adverse outcomes associated with malnutrition.

Recently, several trials have found that nutritional support is beneficial for polymorbid medical patients, resulting in reduced risk for mortality and other complications, as well as improved functionality and quality of life with, however, important heterogeneity among trials.^17^,^19^ Despite these benefits of nutritional support in hospitalised patients, studies focusing on patients with diabetes remain scarce. As a consequence, clinical guidelines on nutrition in patients with diabetes focus mainly on improving glycaemic control through weight loss, healthier eating habits or changing the composition of macronutrients but do not specifically address malnutrition.^2 20 21^ Due to the lack of evidence, malnutrition screening and individualised nutritional interventions are not well established in patients with diabetes in clinical practice, particularly as physicians may be reluctant to use high-energy and protein nutritional support to prevent hyperglycaemia with its negative clinical consequences.

In this study, to investigate the effect of nutritional support on mortality in hospitalised patients with diabetes and nutritional risk, we conducted a preplanned secondary analysis in patients with diabetes participating in the *Effect of early nutritional support on Frailty, Functional Outcomes, and Recovery of malnourished medical inpatients Trial* (EFFORT).^19 22^

## Methods

### Study design and setting

This is a secondary analysis of the prospective, investigator-initiated, randomised controlled Swiss multicentre trial (EFFORT)^19^ that enrolled medical inpatients at nutritional risk. The trial studied the effect of individualised nutritional support compared with usual hospital nutrition on clinical outcomes including mortality in eight secondary and tertiary care hospitals throughout Switzerland. Its protocol, the main results and several secondary analyses have previously been published.^1922^,^28^

### Study population

The initial assessment concerning nutritional risk was done using the Nutritional Risk Screening 2002 (NRS).^29^ The NRS is a validated screening tool to assess the risk of malnutrition in the in-hospital setting. The tool is comprised of several subcategories to which a predefined number of points (0–3) is attributed. These categories are the severity of underlying disease (0–3 points), nutritional status, divided into body mass index (BMI), weight loss and food intake (0–3 points), plus one point for age ≥70 years (maximum seven points). The higher the patients score, the higher the risk for malnutrition. Patients with an NRS total score of 3 or greater that were expected to stay at least 4 days in the hospital were included. Exclusion criteria were an initial hospitalisation in intensive care units or surgical wards; unable to ingest oral nutrition; already receiving nutritional support at admission; with a terminal condition; admitted to hospital because of anorexia nervosa, acute pancreatitis, acute liver failure, cystic fibrosis or stem cell transplantation; after gastric bypass surgery; with contraindications for nutritional support; and previously included in the trial. For this secondary analysis, we included patients with a previous diagnosis of diabetes and those that met the diagnosis of diabetes based on Haemoglobin A1c (HbA1c) value ≥6.5%.^30^

### Study intervention

After inclusion in the trial, patients were randomised 1:1 using an interactive web system. Patients in the intervention group received individualised nutritional support within 48 hours after admission. The nutritional support was based on a previous consensus protocol and in agreement with guidelines for polymorbid patients.^22 31^ A registered, trained dietitian developed a personalised nutritional plan. The daily protein target was set at 1.2–1.5 g/kg of total bodyweight (0.8 g/kg of total bodyweight for patients with renal failure (estimated glomerular filtration rate (eGFR) <30 mL/min)) and the Harris-Benedict equation, adjusted for activity and severity of disease, was used to establish energy requirements.^32^ An oral nutrition plan was developed, which could potentially be extended to enteral tube feeding and parenteral nutrition if the protein and caloric goals were not reached within 5 days. There was no specific protocols regarding nutritional support for patients with diabetes. Nutritional intake was re-assessed every 24–48 hours. Patients in the control group received standard hospital nutrition without nutritional counselling or additional support.

### Endpoints

The primary endpoint for this analysis was all-cause mortality within 30 days, which corresponds to the principal secondary endpoint of the initial EFFORT analysis.^22^ Secondary endpoints included adverse clinical outcomes such as admission to an intensive care unit, non-elective readmission after discharge, length of stay or decline in functional status measured by a decrease of at least 10% as measured by the Barthel index (primary endpoint of the initial trial). Furthermore, we included major complications such as respiratory insufficiency, nosocomial infections, acute kidney injury, gastrointestinal failure and major cardiac events. Safety endpoints were severe hyperglycaemia, occurrence of refeeding syndrome, complications related to enteral or parenteral nutrition, liver or gallbladder dysfunction and adverse gastrointestinal effects. A detailed description of outcomes has been published previously.^22^

Structured follow-up interviews to assess outcomes were conducted by telephone by a blinded study nurse on day 30 and day 180 and annually thereafter. Mortality during follow-up was verified by family members or the patient’s primary care physician.

### Statistical analysis

Continuous variables are expressed as mean±SD; binary and categorical variables as number or count and percentage. Statistical significance was tested at 95% CIs, corresponding to a p value of 0.05.

For the main analysis, we investigated the effect of nutritional support on mortality and other secondary endpoints in patients with diabetes and nutritional risk. We used Cox regressions for all mortality analyses and report HRs, as well as Kaplan-Meier estimates to graphically display survival rates. For other binary endpoints, we used logistic regression models and report ORs. Similar to the initial trial analysis,^19 22^ models were adjusted for predefined covariates including study centre, nutritional risk (based on the NRS total score) and baseline Barthel index. For all subgroup analyses, we calculated an interaction analysis with reporting of p for interaction to understand whether the effect within patients with diabetes would be different from the overall trial population.

In the second step, we investigated the association between diabetes and clinical outcomes in the overall trial population (n=2028). We used the same statistical approach as defined above. Further, we analysed the association of nutritional risk, based on the NRS 2002 score, and long-term mortality within 180 days within the diabetic population.

To understand the effects of nutritional support on glucose control, means of blood glucose values of all patients and each point of measurement were calculated. We had information on available blood glucose data taken as part of standard clinical care of 405 out of 445 patients with diabetes taken on 6 consecutive days. Measurements were done pre-prandially in the morning, at noon and in the evening. There was considerable interpatient variance concerning the number of measurements per patient. Cumulative 6-day mean values were calculated for each patient and each time of measurement. Due to deviation from the normal distribution of blood glucose data, Wilcoxon-Mann-Whitney tests were used to evaluate the differences in glucose serum levels between the intervention and the control groups. We compared the frequency and the rate of hyperglycaemic events between the intervention and the control groups of patients with diabetes. Hyperglycaemia was defined as blood glucose >7 mmol/L.

All statistical analyses were performed with STATA V.17 (Stata Corp, College Station, Texas, USA).

## Results

### Population

Of the 2028 patients included in the original trial, 445 patients had a confirmed diagnosis of diabetes and were considered for this analysis, with 223 randomised to the intervention group and 222 to the control group. The detailed study flow is shown in online supplemental figure 1. The mean age of patients with diabetes was 75.4 (±10.2) years, 61% were male and the mean BMI was 26.9 kg/m^2^ (± 5.7). The main reasons for hospital admission were infection (28%), cancer (16%) and cardiovascular disease (13%). Detailed baseline characteristics of patients with diabetes are presented in table 1. Overall, parameters were evenly distributed between the intervention and the control groups (online supplemental table 1).

**Table 1:** Baseline characteristics stratified according to randomisation

Factor	Control group (n=223)	Intervention group (n=222)	P value
Sociodemographics			
Number	223	222	
Male	131 (59%)	139 (63%)	0.40
Age, mean (SD)	75.3 (10.7)	75.5 (9.6)	0.80
Nutritional assessment			
Mean body mass index (kg/m^2^), mean (SD)	26.9 (5.9)	27.0 (5.5)	0.80
Weight (kg), mean (SD)	76.8 (17.2)	77.0 (17.6)	0.90
Height (cm), mean (SD)	168 (9.1)	168 (9.8)	0.99
NRS total score (%)			
3	63 (28%)	63 (28%)	1.00
4	88 (40%)	91 (41%)	
5	56 (25%)	54 (24%)	
>5	16 (7%)	14 (6%)	
Admission diagnosis			
Infection	71 (32%)	55 (25%)	0.10
Cancer	37 (17%)	35 (16%)	0.81
Cardiovascular disease	30 (14%)	28 (13%)	0.79
Frailty	16 (7%)	17 (8%)	0.85
Lung disease	13 (6%)	6 (3%)	0.10
Gastrointestinal disease	12 (5%)	29 (13%)	0.01
Neurological disease	8 (4%)	7 (3%)	0.80
Renal disease	11 (5%)	15 (7%)	0.41
Metabolic disease	13 (6%)	11 (5%)	0.68
Other	6 (3%)	10 (5%)	0.30
Comorbidity			
Hypertension	168 (75%)	161 (73%)	0.50
Malignant disease	68 (31%)	64 (29%)	0.70
Chronic kidney disease	169 (76%)	172 (78%)	0.67
Coronary artery disease	83 (37%)	77 (35%)	0.58
Congestive heart failure	52 (23%)	42 (19%)	0.3
Chronic obstructive pulmonary disease	32 (14%)	26 (12%)	0.41
Peripheral artery disease	41 (18%)	33 (15%)	0.32
Stroke	27 (12%)	19 (9%)	0.22
Dementia	5 (2%)	11 (5%)	0.12

Table 1: Baseline characteristics of the entire study cohort compared tocompared with the subgroup of patients with diabetes shown according to randomizsation: NRS, .

NRSNutritional Risk Screening 2002

### Association of clinical outcomes and nutritional support in patients with diabetes

**Figure 1:**
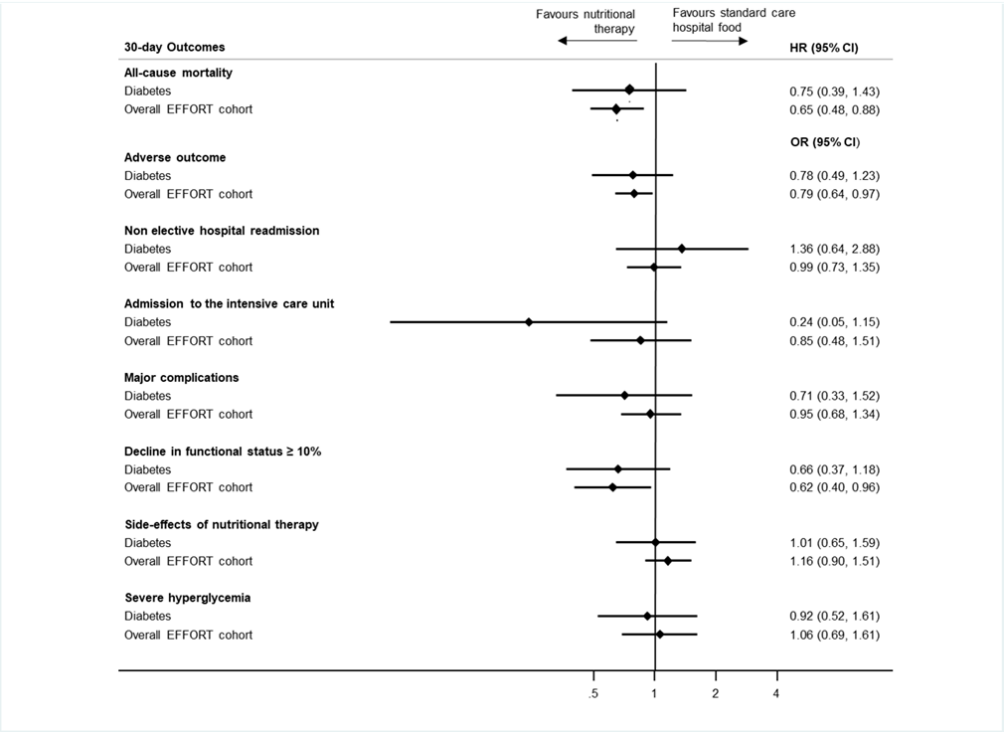
Forest plot showing the effects of nutritional support on clinical outcomes in patients with diabetes and within the overall EFFORT (Effect of early nutritional support on Frailty, Functional Outcomes, and Recovery of malnourished medical inpatients Trial) cohort.

In the next step, we analysed the effect of nutritional support on primary and secondary endpoints in the group of patients with diabetes and compared the results to the overall EFFORT cohort and to patients without diabetes (table 2, figure 1 and online supplemental figure 1). Compared with the control group, the nutritional support group had a lower risk of mortality within 30 days (7% (17/222) vs 10% (24/223), adjusted HR 0.75 (95% CI 0.39 to 1.43)). This effect was similar to the effect in the overall trial with no evidence of a subgroup difference (p=0.92).

**Table 2:** Effect of nutritional support on clinical outcome in patients with diabetes compared with the overall EFFORT cohort

Primary outcomes within 30 days	Events intervention group	Events control group	Adjusted HR (95% CI)	P value	P for interaction
All-cause mortality					
Patients with diabetes	17/222 (7%)	24/223 (10%)	0.75 (0.39 to 1.43)	0.38	0.92
Patient without diabetes	56/793 (7%)	76/790 (10%)	0.80 (0.56 to 1.15)	0.225	
Overall EFFORT cohort	73/1015 (7%)	100/1013 (10%)	0.65 (0.48 to 0.88)	0.006	

Secondary outcomes within 30 days	Events intervention group	Events control group	Adjusted OR(95% CI)	P value	P for interaction
Any adverse outcome					
Patients with diabetes	44/222 (20%)	56/223 (25%)	0.78 (0.49 to 1.23)	0.28	0.66
Patients without diabetes	188/793 (24%)	216/790 (27%)	0.86 (0.69 to 1.09)	0.22	
Overall EFFORT cohort	232/1015 (23%)	272/1013 (27%)	0.79 (0.64 to 0.97)	0.02	
Admission to the intensive care unit					
Patients with diabetes	3/222 (1%)	8/223 (4%)	0.24 (0.05 to 1.15)	0.07	0.15
Patients without diabetes	20/793 (3%)	18/790 (2%)	1.36 (0.69 to 2.67)	0.38	
Overall EFFORT cohort	23/1015 (2%)	26/1013 (3%)	0.85 (0.48 to 1.51)	0.58	
Decline in functional status*					
Patients with diabetes	6/206 (3%)	11/199 (6%)	0.66 (0.37 to 1.18)	0.16	0.75
Patients without diabetes	81/793 (10%)	110/790 (14%)	0.71 (0.52 to 0.97)	0.03	
Overall EFFORT cohort	35/942 (4%)	55/913 (6%)	0.62 (0.40 to 0.96)	0.03	
Side effects from nutritional support					
Any side effects					
Patients with diabetes	54/222 (24%)	52/223 (23%)	1.01 (0.65 to 1.59)	0.95	0.68
Patients without diabetes	108/793 (14%)	93/790 (12%)	1.19 (0.87 to 1.62)	0.27	
Overall EFFORT cohort	162/1015 (16%)	145/1013 (14%)	1.16 (0.90 to 1.51)	0.26	
Severe hyperglycaemia					
Patients with diabetes	29/222 (13%)	30/223 (13%)	0.92 (0.52 to 1.61)	0.76	0.64
Patients without diabetes	19/793 (2%)	16/790 (2%)	1.13 (0.57 to 2.25)	0.71	
Overall EFFORT cohort	48/1015 (5%)	46/1013 (5%)	1.06 (0.69 to 1.61)	0.80	
Refeeding syndrome					
Patients with diabetes	27/222 (12%)	18/223 (8%)	1.52 (0.80 to 2.89)	0.20	0.31
Patients without diabetes	59/793 (7%)	55/790 (7%)	1.07 (0.72 to 1.59)	0.73	
Overall EFFORT cohort	86/1015 (8%)	73/1013 (7%)	1.21 (0.86 to 1.70)	0.27	

Analysis adjusted for NRS, study centre and baseline Barthel index.

* Defined as decline in Barthel index ≥10%.

EFFORTEffect of early nutritional support on Frailty, Functional Outcomes, and Recovery of malnourished medical inpatients TrialNRSNutritional Risk Screening 2002

Nutritional support also improved several secondary endpoints including lower risk for adverse outcomes (20% vs 25%, adjusted OR 0.78 (95% CI 0.49 to 1.23), p=0.28) with no evidence for interaction (p=0.66).

### Side effects of nutritional therapy in patients with diabetes

Finally, we investigated the risk of side effects associated with nutritional support in patients with diabetes. Overall, 24% of patients randomised in the intervention group experienced side effects compared with 23% of patients in the control group (adjusted OR 1.01 (95% CI 0.65 to 1.59), p=0.95). The most common side effects were severe hyperglycaemia and refeeding syndrome. Severe hyperglycaemia occurred in 13% of patients in both the control and the intervention groups with no difference among groups (adjusted OR 0.92 (95% CI 0.52 to 1.61), p=0.76). Similarly, refeeding syndrome occurred in 12% of patients in the intervention group and 8% in the control group, with no significant difference between groups (adjusted OR 1.52 (95% CI 0.80 to 2.89), p=0.20). Results are shown in figure 1 and online supplemental table 2.

**Figure 2:**
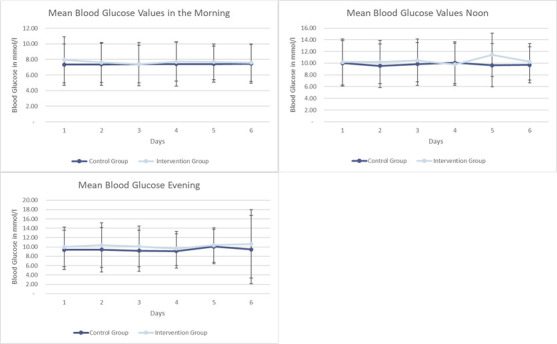
Mean blood glucose values in patients with diabetes according to randomisation. Blood glucose results of patients for 6 consecutive days according to randomisation. SD is indicated.

**Figure 3:**
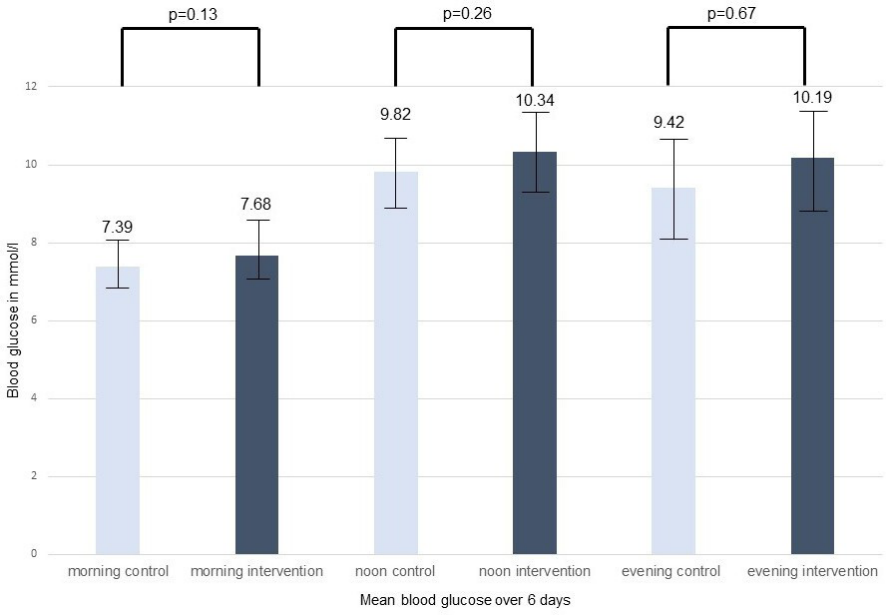
Pooled mean blood glucose values in patients with diabetes according to randomisation.

To further examine glycaemic control between patients with diabetes in the intervention and control groups, we analysed the mean serum blood glucose levels measured during the hospital stay as usual care. As shown in figures 2 and 3, glucose levels in the first 6 days, as well as the pooled means over 6 days of hospitalisation, were similar in the intervention and control groups. Boxplots of glucose measures can be found in online supplemental table 3 and online supplemental figure 3. Comparing hyperglycaemic events, we found similar rates of hyperglycaemia in both the control and the intervention groups of patients with diabetes (online supplemental figure 4).

### Association of diabetes with clinical outcomes

In the first step, we analysed the association between diabetes and clinical outcomes using unadjusted and adjusted regression models within the overall EFFORT patient cohort (n=2028). Regarding the primary endpoint, diabetes was not associated with 30-day mortality (HR 1.10, 95% CI 0.78 to 1.56, p=0.59). However, diabetes was associated with several secondary endpoints including risk of severe in-hospital hyperglycaemia (OR 6.76, 95% CI 4.39 to 10.42, p<0.001), risk of refeeding syndrome (OR 1.45, 95% CI 1.01 to 2.08, p=0.04), 180-day mortality (OR 1.33, 95% CI 1.04 to 1.68, p=0.02) and length of hospital stay (mean difference 0.71, 95% CI 0.02 to 1.40, p=0.04). After adjustment for nutritional risk, baseline Barthel index and centre of admission, there was still a statistically significant association between diabetes severe hyperglycaemia (adjusted OR 6.82, 95% CI 4.39 to 10.59, p<0.001). More detailed results can be found in online supplemental tables 4 and 5.

### Association of nutritional risk severity and mortality among patients with diabetes

Next, we investigated the association of severity of nutritional risk, indicated by the NRS total score, and mortality over 180 days among patients with diabetes. There was an increased risk for mortality with increasing nutritional risk. Compared with patients with moderate nutritional risk (NRS total score of 3 points), patients with higher nutritional risk showed an increased risk for 180-day mortality (HR 1.56 (95% CI 0.96 to 2.57), p=0.08, HR 2.22 (95% CI 1.32 to 3.73), p<0.01 and HR 2.80 (95% CI 1.42 to 5.52)), p<0.01 for NRS score of 4, 5 and 6) (online supplemental figure 5)

## Discussion

There are two key findings from this secondary analysis of a Swiss multicentre, randomised trial focusing on the effect of nutritional support among patients with diabetes and at nutritional risk. First, we found that compared with patients without diabetes, patients with diabetes had a higher risk for mortality and adverse clinical outcomes also including diabetes-specific outcomes such as hyperglycaemia. Second, regarding the effects of our intervention, we found a mortality benefit of nutritional support compared with usual care nutrition in malnourished patients with diabetes, which was similar to the significant benefit observed in the overall malnourished medical inpatient population. Importantly, there was no increased risk of hyperglycaemia and other adverse outcomes associated with nutritional support in patients with diabetes suggesting that nutrition appears safe regarding glycaemic control.

There is a lack of strong guideline recommendation regarding the use of nutritional support in hospitalised patients with diabetes and increased malnutrition risk from diabetic societies.^2 30^ This is arguably a result of the lack of outcome data proving that nutritional support in fact improves outcomes without worsening the glycaemic control of patients. Here, despite the limitations of a secondary analysis, our data provide important insights suggesting that indeed patients with diabetes benefit from nutritional screening and start of nutritional therapy if increased risk is detected. This approach is also supported by the current uropean Society for Parenteral and Enteral Nutrition (ESPEN) recommendations suggesting that malnutrition in the elderly should be treated with nutritional support, even if the patient has diabetes, because the negative consequences of malnutrition may outweigh the negative effects of nutritional support.^33 34^ ESPEN guidelines highlight that in elderly patients with diabetes with a high risk for frailty, sarcopenia or osteopenia, it is particularly important to ensure sufficient nutritional intake to prohibit further functional decline.^35^

In our cohort of mostly elderly and multimorbid patients with a mean age of 75 years, the prevalence of diabetes was 20% and thus highly prevalent. This corresponds to reported prevalences of diabetes in the age group of people between 65 and 80 years according to the International Diabetes Federation.^11 12^ We also found a high prevalence of comorbidities in our cohort, especially for cardiovascular diseases such as hypertension, kidney disease or coronary heart disease, which again is in line with other studies.^12 14^ Given the demographic changes, we expect thus that the number of multimorbid patients with diabetes being treated in hospital will further increase, underscoring the importance of understanding how to best manage malnutrition risks in this patient population.

In our cohort of inpatients with diabetes and several other acute and chronic diseases, severe hyperglycaemia was frequent.^36 37^ Furthermore, chronic glycaemic control was suboptimal in several patients, as shown by an HbA1c >7%, a marker that is not affected by the presence of acute illness.^38^ However, blood glucose levels may continue to rise in the context of stress hyperglycaemia and increased inflammation, which is an independent risk factor for adverse clinical outcome in the intensive care unit.^39 40^ Additionally, commonly prescribed oral anti-diabetic drugs in the outpatient setting, for example, metformin or sulfonylureas, are often discontinued during hospitalisation. Importantly, however, nutritional support did not contribute to the risk of hyperglycaemia, and glucose levels were similar in both groups during the hospital stay. Based on these observations, malnutrition therapy in acutely ill patients with diabetes should not be considered as a risk factor for diabetic complications but as a factor contributing to recovery from disease. In particular, in patients with kidney disease, a well-known complication of diabetes, nutritional therapy has been shown to be highly beneficial for patients, which may have influenced the results in our population.^25^

### Strengths and limitations

Our study has several strengths and limitations. The topic of malnutrition in the patient with diabetes is important since both diabetes and malnutrition have high prevalences among the increasingly ageing population. In addition to many observational studies, this is, to our knowledge, the first analysis looking at the effect of nutritional support on clinical outcomes using data from a large multicentre randomised trial, which provides high-quality data. Still, as a secondary analysis, this study is rather hypothesis-generating and needs validation in further prospective studies. Also, we did not systematically collect blood glucose values in all patients but relied on measurements taken during clinical routine, resulting in a large variance between patients in the number of measurements taken. Furthermore, the nutritional supplement used was a standard formula and not a diabetes-specific formula with less carbohydrates. Recently, there has been some evidence that the use of diabetes-specific formulas which tend to be lower in carbohydrates with a high glycaemic index (GI), enriched with fibre, and high in fats, especially monounsaturated fatty acids,^41^ may be beneficial for patients with diabetes in terms of glycaemic control, both in the long term, as measured by HbA1c, and in the short term, measuring post-prandial peak values.^42 43^ Furthermore, the type of anti-diabetic drugs such as GLP-1 receptor agonist which affect appetite and, thus, nutritional intake was not considered in this study. Additionally, our study population was relatively small compared with the total cohort, resulting in lower statistical power when investigating effects on outcomes. Furthermore, the effect of long-term nutritional support on glycaemic control or clinical outcomes cannot be derived from this analysis as we only provided nutritional support during hospital stay. Thus, this secondary analysis should be viewed as exploratory rather than definite.

## Conclusion

Our secondary analysis revealed no significant impact on mortality from individualised nutritional support in patients with diabetes, nor did it indicate any diabetes-associated side effects. Clearly, more research in this field is needed to validate the results to change the clinical practice of nutritional therapy in patients with diabetes and malnutrition.

## supplementary material

10.1136/bmjopen-2024-084754 online supplemental file 1

## Data Availability

Data described in the manuscript, code book, and analytic code will be made available to others with the publication of this manuscript, as already outlined in the primary EFFORT publication, on receipt of a letter of intention detailing the study hypothesis and statistical analysis plan. A signed data access agreement is required from all applicants. Please send requests to the principal investigator of this trial. There is no additional data available.
