# Supplementary material for: Nutritional support in hospitalised patients with diabetes and risk for malnutrition: a secondary analysis of an investigator-initiated, Swiss, randomised controlled multicentre trial
Source: BMJ Open. 2024 Aug 16;14(8):e084754. doi: 10.1136/bmjopen-2024-084754 (PMC11535698; doi:10.1136/bmjopen-2024-084754)
Supplement: online supplemental file 1 [file bmjopen-14-8-s001.pdf]

# Appendix:

## Nutritional support in hospitalized patients with diabetes and risk for malnutrition

***A secondary analysis of an investigator initiated, Swiss, randomized controlled multi-center trial***

Bettina Keller [1,2]*, Carla Wunderle[1]*, Pascal Tribolet [1,3,4], Zeno Stanga [5], Nina Kaegi-Braun[6], Beat Mueller [1,2,] and Philipp Schuetz[1,2]

1 Medical University Department, Division of General Internal and Emergency Medicine, Kantonsspital Aarau, Aarau, Switzerland

2 Medical Faculty of the University of Basel, Basel, Switzerland

3 Department of Health Professions, Bern University of Applied Sciences, Bern, Switzerland

4 Faculty of Life Sciences University of Vienna, Vienna, Austria

5 Division of Diabetes, Endocrinology, Nutritional Medicine and Metabolism, Bern University Hospital and University of Bern, Bern, Switzerland

6 Department of Bioscience and Nutrition, Karolinska Institutet, 17177 Stockholm, Sweden

**Supplemental Figure 1: Trial Flow Chart**

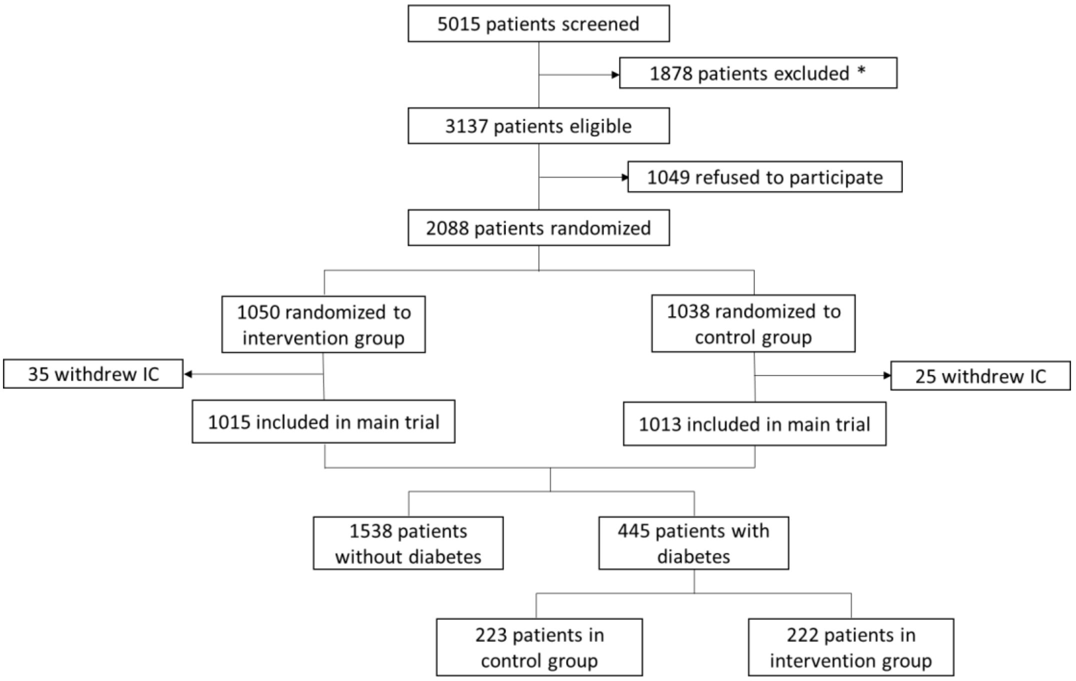


*Figure 1: Study flow chart. Abbreviations: IC; informed consent. * Reasons for exclusion: 145 surgical patients, 268 unable to ingest oral nutrition, 158 with a terminal condition, 719 patients already receiving nutritional therapy upon admission, 31 hospitalized because of anorexia nervosa, 161 with acute pancreatitis, 81 with acute liver failure, 6 with cystic fibrosis, 11 with stem cell transplantation, 27 with malnutrition after gastric bypass surgery, 43 with contradiction against nutritional therapy and 228 previously included in the trial.*

**Supplemental Figure 2: 30-day Kaplan-Meier survival estimates**

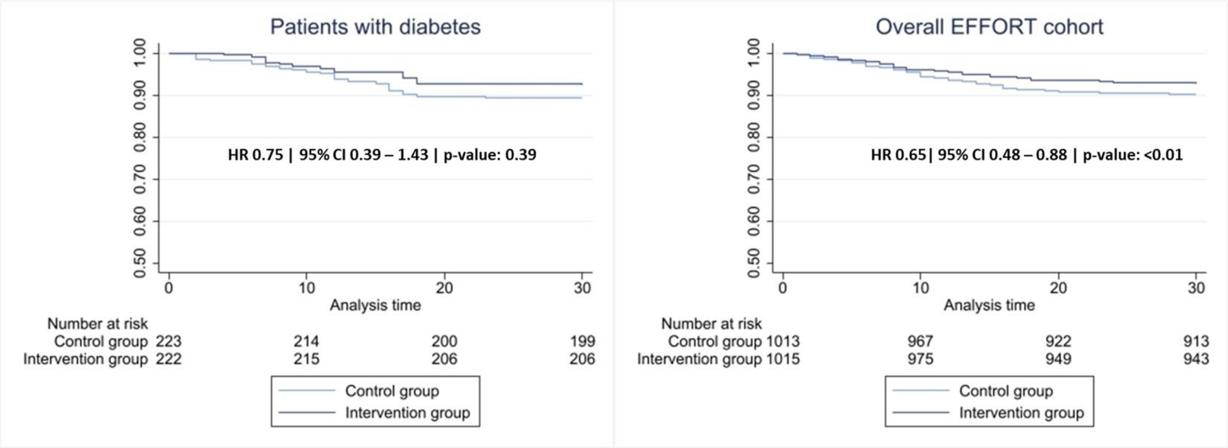


*Figure 2: Survival analysis in patients with diabetes (left) and the total cohort (right): HR, Hazard ratio; CI, Confidence interval*

**Supplemental Figure 3: Mean blood glucose values**

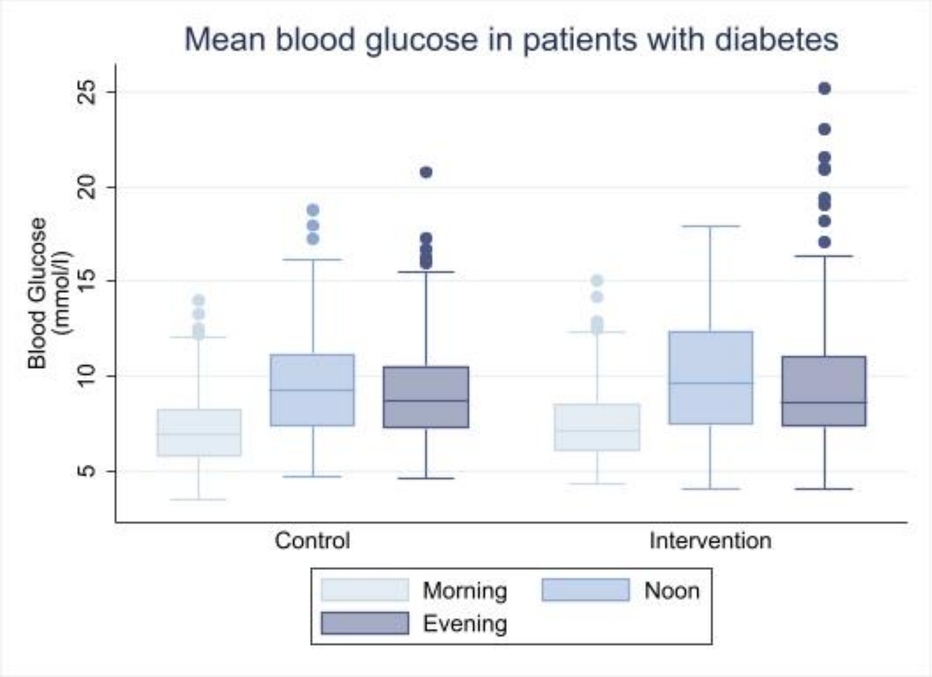


*Figure 4: Box plot illustrating mean glucose values of patients with diabetes according to randomization. (Whiskers indicating 25% and 75% of values, median indicated within the box)*

**Supplemental Figure 4: Mean rate of hyperglycemias in patients with diabetes**

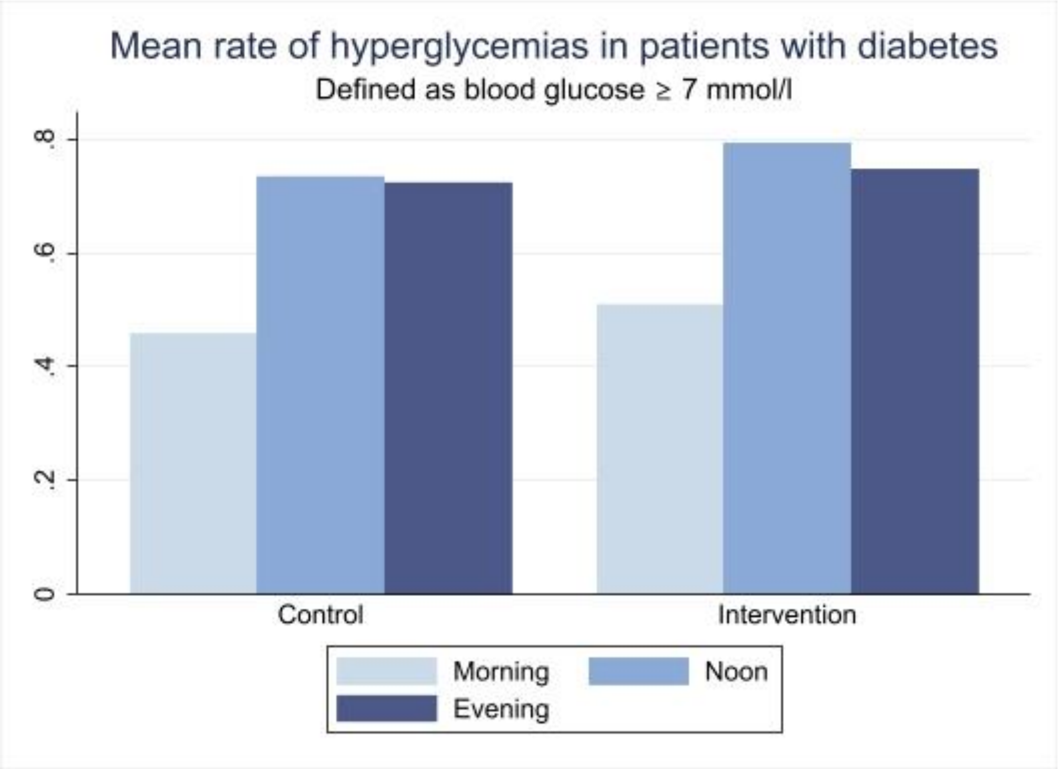


*Figure 3: Bar graph illustrating the rates of hyperglycemia in patients with diabetes according to randomization.*

**Supplemental Figure 5: 180-day Kaplan-Meier survival estimates for patients with diabetes according to NRS total score**

*NRS, Nutritional Risk Screening 2002*

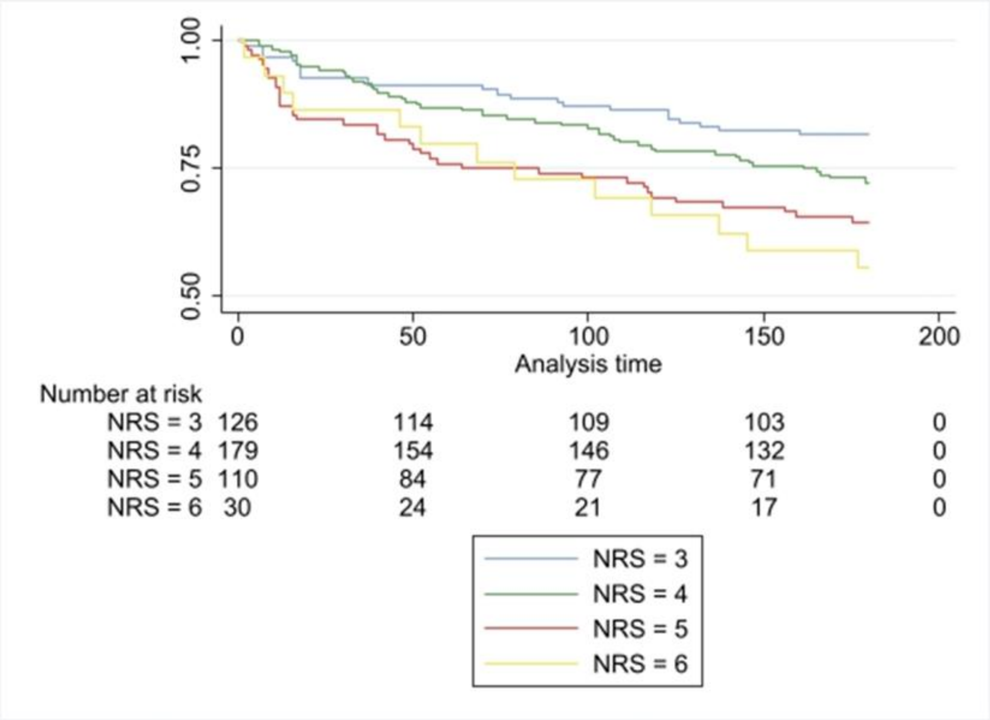

**Supplemental Table 1: Baseline characteristics of the total EFFORT cohort compared to patients with diabetes stratified by randomization**

| Factor | Total cohort (n=2,028) | Patients with diabetes control group (n=223) | Patients with diabetes intervention group (n=222) | p-value |
|---|---|---|---|---|
| **Sociodemographics** | | | | |
| **Number** | 2,028 | 223 | 222 | |
| **Male** | 1064 (52%) | 131 (59%) | 139 (63%) | 0.40 |
| **Age, mean (SD)** | 72. 6 (14.1) | 75.3 (10.7) | 75.5 (9.6) | 0.80 |
| **Nutritional assessment** | | | | |
| **Mean body-mass-index (kg/$m^2$), mean (SD)** | 24.8 (5.3) | 26.9 (5.9) | 27.0 (5.5) | 0.80 |
| **Weight (kg), mean (SD)** | 70.9 (16.7) | 76.8 (17.2) | 77.0 (17.6) | 0.90 |
| **Height (cm), mean (SD)** | 168 (9.3) | 168 (9.1) | 168 (9.8) | 0.99 |
| **NRS total score (%)** | | | | |
| **3** | 624 (31%) | 63 (28%) | 63 (28%) | 1.00 |
| **4** | 775 (38%) | 88 (40%) | 91 (41%) | |
| **5** | 524 (26%) | 56 (25%) | 54 (24%) | |
| **>5** | 105 (5%) | 16 (7%) | 14 (6%) | |
| **Admission diagnosis** | | | | |
| **Infection** | 613 (30%) | 71 (32%) | 55 (25%) | 0.10 |
| **Cancer** | 374 (18%) | 37 (17%) | 35 (16%) | 0.81 |
| **Cardiovascular disease** | 205 (10%) | 30 (14%) | 28 (13%) | 0.79 |
| **Frailty** | 194 (10%) | 16 (7%) | 17 (8%) | 0.85 |
| **Lung disease** | 125 (6%) | 13 (6%) | 6 (3%) | 0.10 |
| **Gastrointestinal disease** | 164 (8%) | 12 (5%) | 29 (13%) | 0.01 |
| **Neurological disease** | 95 (5%) | 8 (4%) | 7 (3%) | 0.80 |
| **Renal disease** | 68 (3%) | 11 (5%) | 15 (7%) | 0.41 |
| **Metabolic disease** | 62 (3%) | 13 (6%) | 11 (5%) | 0.68 |
| **Other** | 55 (3%) | 6 (3%) | 10 (5%) | 0.30 |
| **Comorbidity** | | | | |
| **Hypertension** | 1109 (55%) | 168 (75%) | 161 (73%) | 0.50 |
| **Malignant disease** | 667 (33%) | 68 (31%) | 64 (29%) | 0.70 |
| **Chronic kidney disease** | 1347 (66%) | 169 (76%) | 172 (78%) | 0.67 |
| **Coronary artery disease** | 566 (28%) | 83 (37%) | 77 (35%) | 0.58 |
| **Congestive heart failure** | 353 (17%) | 52 (23%) | 42 (19%) | 0.3 |
| **Chronic obstructive pulmonary disease** | 303 (15%) | 32 (14%) | 26 (12%) | 0.41 |
| **Peripheral artery disease** | 186 (9%) | 41 (18%) | 33 (15%) | 0.32 |
| **Stroke** | 162 (8%) | 27 (12%) | 19 (9%) | 0.22 |
| **Dementia** | 75 (4%) | 5 (2%) | 11 (5%) | 0.12 |

*Table 1: Baseline characteristics of the entire study cohort compared to the subgroup of patients with diabetes shown according to randomization: NRS, Nutritional Risk Screening 2002.*

**Supplemental Table 2: Multivariate analysis of clinical outcomes and randomization for patients with diabetes (Extension)**

| Primary outcomes within 30 days | Events intervention group | Events control group | Adjusted OR or coefficient (95% CI) | p-value | p for inter-action |
|---|---|---|---|---|---|
| **Major complications** | | | | | |
| **Any major complications** | | | | | |
| **Patients with diabetes** | 13/222 (6%) | 17/223 (8%) | 0.71 (0.33 - 1.52) | 0.37 | 0.46 |
| **Patients without diabetes** | 61/793 (8%) | 59/790 (7%) | 1.03 (0.70 – 1.51) | 0.88 | |
| **Overall EFFORT cohort** | 74/1015 (7%) | 76/1013 (8%) | 0.95 (0.68 - 1.34) | 0.79 | |
| **Respiratory failure** | | | | | |
| **Patients with diabetes** | 3/222 (1%) | 3/223 (1%) | 0.70 (0.11-4.27) | 0.70 | 0.93 |
| **Patients without diabetes** | 11/793 (1%) | 10/790 (1%) | 1.24 (0.51 – 3.03) | 0.64 | |
| **Overall EFFORT cohort** | 14/1015 (1%) | 13/1013 (1%) | 1.06 (0.49 - 2.28) | 0.89 | |
| **Nosocomial infection** | | | | | |
| **Patients with diabetes** | 9/222 (4%) | 8/223 (4%) | 1.03 (0.38 - 2.81) | 0.95 | 0.82 |
| **Patients without diabetes** | 31/793 (4%) | 31/790 (4%) | 0.93 (0.55 – 1.58) | 0.80 | |
| **Overall EFFORT cohort** | 40 /1015 (4%) | 39/1013 (4%) | 1.01 (0.63 - 1.59) | 0.98 | |
| **Major cardiovascular events** | | | | | |
| **Patients with diabetes** | 2/222 (1%) | 2/223 (1%) | 1.12 (0.15 - 8.49) | 0.91 | 0.88 |
| **Patients without diabetes** | 6/793 (1%) | 5/790 (1%) | 1.53 (0.43 – 5.47) | 0.51 | |
| **Overall EFFORT cohort** | 8 /1015 (1%) | 7/1013 (1%) | 1.11 (0.40 - 3.11) | 0.84 | |
| **Acute kidney injury** | | | | | |
| **Patients with diabetes** | 7/222 (3%) | 12/223 (5%) | 0.47 (0.17 - 1.29) | 0.14 | 0.15 |
| **Patients without diabetes** | 25/793 (3%) | 19/790 (2%) | 1.30 (0.70 – 2.40) | 0.40 | |
| **Overall EFFORT cohort** | 32/1015 (3%) | 31/1013 (3%) | 1.01 (0.61 - 1.69) | 0.96 | |
| **Gastrointestinal events** | | | | | |
| **Patients with diabetes** | 1/222 (<1%) | 2/223 (1%) | 0.46 (0.04 - 5.26) | 0.54 | 0.88 |
| **Patients without diabetes** | 8/793 (1%) | 13/790 (2%) | 0.67 (0.27 – 1.65) | 0.38 | |
| **Overall EFFORT cohort** | 9/1013 (1%) | 15/1013 (1%) | 0.57 (0.25 - 1.31) | 0.19 | |
| **Side effects from nutritional therapy** | | | | | |
| **Gastrointestinal side-effects** | | | | | |
| **Patients with diabetes** | 8/222 (4%) | 7/223(3%) | 1.05 (0.35 - 3.19) | 0.93 | 0.88 |
| **Patients without diabetes** | 35/793 (4%) | 33/790 (4%) | 1.15 (0.68 – 1.95) | 0.60 | |
| **Overall EFFORT cohort** | 43/1015 (4%) | 40/1013 (4%) | 1.12 (0.68 - 1.83) | 0.66 | |
| **Complications due to enteral feeding or parenteral nutrition** | | | | | |
| **Patients with diabetes** | 1/222 (<1%) | 2/223 (1%) | 0.48 (0.04 - 5.49) | 0.55 | 0.21 |
| **Patients without diabetes** | - | - | - | - | |
| **Overall EFFORT cohort** | 5/1015 (<1%) | 3/1013 (<1%) | 1.63 (0.38 - 6.95) | 0.51 | |
| **Liver or gallbladder dysfunction** | | | | | |
| **Patients with diabetes** | 2/222 | 4/223 (2%) | 0.20 (0.02 - 1.97) | 0.17 | 0.82 |

| | | | | | |
|---|---|---|---|---|---|
| | (1%) | | | | |
| **Patients without diabetes** | 2/793 (<1%) | 3/790 (<1%) | 0.69 (0.11 – 4.17) | 0.69 | |
| **Overall EFFORT cohort** | 4/1015 (<1%) | 7/1013 (1%) | 1.63 (0.38 - 6.95) | 0.51 | |
| **Mean length of stay (d)** | | | | | |
| **Patients with diabetes** | 10.4 | 9.8 | 0.49 (-0.67 - 1.66) | 0.41 | 0.18 |
| **Patients without diabetes** | 9.2 | 9.6 | -0.53 (-1.17 – 0.12) | 0.11 | |
| **Overall EFFORT cohort** | 9.5 | 9.6 | -0.21 (-0.76 - 0.35) | 0.46 | |

*Table 4: Multivariate analysis, adjusted for NRS, Center of admission and Barthel index, examining the association between nutritional support and clinical outcomes: NRS, Nutritional Risk Screening 2002*

**Supplemental Table 3: Mean blood glucose values in patients with diabetes**

| | Control group | | | Intervention group | | |
|---|---|---|---|---|---|---|
| | **Morning** | **Noon** | **Evening** | **Morning** | **Noon** | **Evening** |
| **Day 1** | 7.34 | 10.01 | 9.37 | 7.95 | 10.21 | 10.01 |
| **Day 2** | 7.36 | 9.57 | 9.42 | 7.61 | 10.22 | 10.38 |
| **Day 3** | 7.41 | 9.86 | 9.18 | 7.42 | 10.47 | 10.13 |
| **Day 4** | 7.41 | 10.08 | 9.11 | 7.71 | 9.80 | 9.67 |
| **Day 5** | 7.40 | 9.65 | 10.07 | 7.69 | 11.43 | 10.38 |
| **Day 6** | 7.45 | 9.71 | 9.45 | 7.58 | 10.24 | 10.66 |

**Supplemental Table 4: Univariate analysis of the association of diabetes and outcomes**

| Outcomes | Hazard ratio , coefficient or odds ratio | 95% Confidence Interval | p-value |
|---|---|---|---|
| **Survival analysis** | | | |
| **30-day mortality** | 1.10 | 0.78-1.56 | 0.59 |
| **180-day mortality** | 1.25 | 1.02-1.54 | 0.03 |
| **3-year mortality** | 1.14 | 0.98-1.33 | 0.08 |
| **Readmission rates** | | | |
| **30-day non-elective readmission** | 0.69 | 0.46-1.04 | 0.07 |
| **180-day non-elective readmission** | 0.92 | 0.73-1.17 | 0.51 |
| **5-year non-elective readmission** | 0.90 | 0.64-1.27 | 0.56 |
| **Major adverse outcomes within 30 days** | | | |
| **Major complications** | 0.88 | 0.58-1.33 | 0.55 |
| **Admission to intensive care unit** | 1.03 | 0.52-2.03 | 0.93 |
| **Nosocomial infection** | 0.97 | 0.56-1.68 | 0.93 |
| **Respiratory failure** | 1.02 | 0.41-2.53 | 0.97 |
| **Gastrointestinal events** | 0.50 | 0.15-1.70 | 0.27 |
| **Acute kidney injury** | 1.56 | 0.90-2.70 | 0.11 |
| **Major cardiovascular events** | 1.30 | 0.41-4.09 | 0.66 |
| **Functional decline ≥ 10%*** | 1.03 | 0.75-1.42 | 0.87 |
| **Any adverse outcome** | 0.85 | 0.66-1.09 | 0.19 |
| **Side effects of nutritional support** | | | |
| **Any side-effects** | 2.15 | 1.65-2.80 | <0.01 |
| **Gastrointestinal side effects** | 0.78 | 0.44-1.37 | 0.39 |
| **Severe hyperglycemia** | 6.76 | 4.39-10.42 | <0.01 |
| **Liver or gallbladder dysfunction** | 4.31 | 1.31-14.20 | 0.02 |
| **Refeeding Syndrom** | 1.45 | 1.01-2.08 | 0.04 |
| **Feeding tube** | 1.42 | 0.28-7.37 | 0.67 |
| **Parenteral nutrition of feeding tube** | 2.14 | 0.51-9.00 | 0.30 |
| **Length of stay** | 0.71 | 0.02-1.40 | 0.04 |
| **180-day weight change** | -0.94 | -1.71--0.17 | 0.02 |
| **30-day weight change** | -0.08 | -0.75-0.58 | 0.81 |

*Table 1: Univariate regression; association of diabetes with different outcomes. Hazard ratios were reported for survival analyses, odds ratios were reported for binary outcomes, coefficients were reported for continuous outcomes.*

**Defined as decline in Barthel index of ≥10%*

**Supplemental Table 5: Multivariate analysis of the association of diabetes and outcomes**

| **Outcomes** | **Hazard ratio, coefficient or Odds ratio** | **95% Confidence Interval** | **p-value** |
|---|---|---|---|
| **Mortality analysis** | | | |
| **30-day mortality** | 1.05 | 0.73-1.53 | 0.76 |
| **180-day mortality** | 1.21 | 0.98-1.49 | 0.07 |
| **3-year mortality** | 1.10 | 0.94-1.28 | 0.22 |
| **Hospital readmissions** | | | |
| **30-day non-elective readmisssion** | 0.70 | 0.46-1.05 | 0.08 |
| **180-day non-elective readmission** | 0.95 | 0.75-1.21 | 0.69 |
| **5-year non-elective readmission** | 0.95 | 0.67-1.35 | 0.77 |
| **Major complications within 30 days** | | | |
| **Admission to intensive care unit** | 1.01 | 0.50-2.06 | 0.98 |
| **Nosocomial Infection** | 0.95 | 0.54-1.68 | 0.87 |
| **Acute kidney injury** | 1.44 | 0.81-2.54 | 0.21 |
| **Respiratory failure** | 0.85 | 0.31-2.29 | 0.74 |
| **Gastrointestinal events** | 0.52 | 0.15-1.75 | 0.29 |
| **Adverse outcomes** | 0.83 | 0.64-1.07 | 0.16 |
| **Major complications** | 0.84 | 0.55-1.29 | 0.43 |
| **Major cardiovascular event** | 1.27 | 0.39-4.09 | 0.69 |
| **Functional decline ≥ 10%*** | 0.99 | 0.72-1.37 | 0.97 |
| **Side effects from nutritional therapy** | | | |
| **Any side effects** | 2.24 | 1.70-2.95 | <0.01 |
| **Gastrointestinal side effects** | 0.76 | 0.41-1.42 | 0.38 |
| **Severe hyperglycemia** | 6.82 | 4.39-10.59 | <0.01 |
| **Liver or gallbladder dysfunction** | 3.49 | 0.97-12.58 | 0.06 |
| **Refeeding syndrome** | 1.43 | 0.98-2.07 | 0.06 |
| **Feeding tube** | 1.72 | 0.31-9.51 | 0.53 |
| **Parenteral feeding or feeding tube** | 2.66 | 0.59-12.01 | 0.20 |
| **Length of stay** | 0.46 | -0.22-1.14 | 0.18 |
| **180-day weight change** | -0.94 | -1.71--0.16 | 0.02 |
| **30-day weight change** | -0.09 | -0.76-0.58 | 0.80 |

Table 2: Multivariate regression adjusted for Nutritional Risk Screening 2002, Barthel Index and center of admission, association of diabetes with different outcomes. *Hazard ratios were reported for survival analyses, odds ratios were reported for binary outcomes, coefficients were reported for continuous outcomes.*

*Defined as decline in Barthel index of ≥10%

**Full list of investigators involved in the EFFORT trial:**

- Kantonsspital Aarau: Thomas Baumgartner;  Valerie Bächli; Luca Bernasconi; Lisa Bonoure; Manuela Deiss; Andreas Eckart; Rebecca Fehr, Susan Felder; Natalie Friedli; Martina Geiser, Filomena Gomes, Lena Grädel; Andreas Huber; Daniel Koch; Alexander Kutz; Svenja Laukemann; Olivia Neeser; Manuela Nickler; Marc Meier; Marc Meili; Beat Mueller; Jonas Odermatt; Manuel Ottiger; Isabel Pulvermüller; Anna Christina Rast; Katharina Regez; Ramon Sager; Diana Sbiti; Ursula Schild; Philipp Schuetz; Deborah Steiner; Alaadin Vögeli; Yannick Wirz; Esther Wyrsch; Giedre Zurauskaite; Seline Zurfluh;
- Inselspital Bern: Evelyne Abgottspon; Drahomir Aujesky; Adrian Baumann; Kathrin Blaser; Jacques Donzé, Tanja Flückiger; Cindy Groen; Daniela Häfeli; Corinne Hänzi; Katrin Lengacher; Melisa Merdanovic; Sarah Pfaffen; Nicolas Rodondi; Nathalie Schwab; Zeno Stanga; Monica von Brevern; Sophie von Lerber;
- Spital Lachen: Thomas Bregenzer; Bruno Schiesser; Pascal Tribolet; Anita Wild;
- Luzerner Kantonsspital: Daniela Buhl, Christoph Henzen; Silvia Mattmann; Melina Nigg; Sara Ramseier; Michael Trummler
- Kantonsspital Münsterlingen: Nina Braun; Claus Hoess, Cornelia Ebneter; Dominique Mannhart; Vojtech Pavlicek, Sarah Schmid
- Bürgerspital Solothurn: Cornelia Albrecht; Claudia Brand; Katharina Ilic; Lisa Tanner; Robert, Thomann; Rahel von Felten;
- Kantonsspital St.Gallen: Carmen Benz; Michael Brändle, Stefan Bilz; Madlaina Höhener; Sarah Sigrist; Rahel Stadler; Alexandra Wick
- Kantonsspital Baselland, Bruderholz: Jonas Rutishauser; Marianne Waldmeier
