# Supplementary material for: Nutritional support in hospitalised patients with diabetes and risk for malnutrition: a secondary analysis of an investigator-initiated, Swiss, randomised controlled multicentre trial
Source: BMJ Open. 2024 Aug 16;14(8):e084754. doi: 10.1136/bmjopen-2024-084754 (PMC11535698; doi:10.1136/bmjopen-2024-084754)
Supplement: Supplementary file 2 [file bmjopen-2024-084754.reviewer_comments.pdf]

## PEER REVIEW HISTORY

BMJ Open publishes all reviews undertaken for accepted manuscripts. Reviewers are asked to complete a checklist review form and are provided with free text boxes to elaborate on their assessment. These free text comments are reproduced below.

## ARTICLE DETAILS

| | |
|---|---|
| **TITLE (PROVISIONAL)** | Nutritional support in hospitalized patients with diabetes and risk for malnutrition A secondary analysis of an investigator initiated, Swiss, randomized controlled multi-center trial |
| **AUTHORS** | Keller, Bettina; Wunderle, Carla; Tribolet, Pascal; Stanga, Zeno; Kaegi-Braun, Nina; Mueller, Beat; Schuetz, Philipp |

## VERSION 1 - REVIEW

| | |
|---|---|
| **REVIEWER NAME** | ***Boersma, Eric*** |
| **REVIEWER AFFILIATION** | Thoraxcenter, Erasmus MC, Dept. of Cardiology |
| **REVIEWER CONFLICT OF INTEREST** | None |
| **DATE REVIEW RETURNED** | 23-Feb-2024 |

| | |
|---|---|
| **GENERAL COMMENTS** | In the EFFORT randomised trial, Lancet 2019, the authors studied the effect of nutritional support in medical inpatients at nutritional risk. Nutritional support appeared associated with a reduced incidence of any 'adverse clinical outcome' at 30 days (primary endpoint) as compared with 'standard hospital nutrition'. The adjusted odds ratio (OR) was 0·79 (95% CI 0·64-0·97). Also all-cause death was significantly reduced, with OR 0.65 (95% CI 0.47-0.91). The effect of the intervention on the primary endpoint in subjects with diabetes was reported in the main results paper (doi 10.1016/S0140-6736(18)32776-4), but the effect on mortality was not reported. The current paper presents a further analysis of the EFFORT trial, focussing on modifications of the effect of nutritional support on mortality between patients with versus without diabetes. There appeared no evidence of effect modification.<br><br>From methodological/statistical point of view I have the following questions and remarks:<br><br>1. Using the EFFORT data, the authors studied a) the association between diabetes and clinical outcomes, and b) the effect of randomisation to nutritional support (versus) in patients with diabetes. These are separate research questions, which need to be clearly distinguished. Also, the statistical methods to address these questions differ, e.g. with respect to the adjustment of potential confounders (and the selection of these confounders). The (current description of the) applied statistical methods fails to make this distinction and needs to be modified.<br><br>2. In view of the research goal, the specific attention (in the Results section) for the relation between nutritional severity risk and outcome is not clear (and is not addressed as such in the Methods section). It is also not clear why this analysis is limited to patients with diabetes only. |

| | |
|---|---|
| | 3. The authors state (Abstract and Methods) that the primary endpoint of EFFORT was 30-day all-cause mortality. However, the main results paper (see above) states that the primary endpoint was a composite of any 'adverse clinical outcome(s)', defined as 'all-cause mortality, admission to intensive care, non-elective hospital readmission, major complications, and decline in functional status at 30 days'. The findings with regard to mortality as a single endpoint should be interpreted in that context.<br><br>4. The authors use a mixture of Cox regression for 'time-to-event analyses' (hazard ratios [HR] are reported) and logistic regression for 'binary outcomes' (ORs are reported). However, the 'binary outcomes' occur during follow-up. Based on what criteria did the authors decide to use Cox versus logistic?. Note that the main results paper presents treatment effects in terms of odds ratios.<br><br>5. (How) did the authors evaluate the proportional hazard (PH) assumption with respect to the Cox model? And was the PH assumption satisfied?<br><br>6. 'Continuous variables are expressed as mean ± standard deviation (SD) …' (How) did the authors evaluate normality?<br><br>7. Regarding the effect of the intervention, the authors report HRs and ORs for patients with diabetes and for the overall cohort. They should also report estimates in patients without diabetes for comparison (see e.g. Table 2).<br><br>8. The authors state (Abstract) that '… there was a 25%-reduction in the risk of mortality in patients with diabetes receiving nutritional support compared to controls (7% vs. 10%, adjusted HR 0.75, [95% CI 0.39 to 1.43]) …'. However, factually, a) this mortality reduction in the sub-population of patients with diabetes is not statistically significant, and b) there was no evidence of interaction. Hence, the best estimate of treatment effect is based on the entire sample of patients with and without diabetes.<br><br>9. The authors state 'In a first step, we analyzed the association between diabetes and clinical outcomes using unadjusted and adjusted regression models within the overall EFFORT patient cohort (n=2028).' Was randomly allocated treatment included as a determinant of outcome in these models? |

| | |
|---|---|
| **REVIEWER NAME** | ***Viana, Marina Verçoza*** |
| **REVIEWER AFFILIATION** | Hospital de Clínicas de Porto Alegre |
| **REVIEWER CONFLICT OF INTEREST** | none |
| **DATE REVIEW RETURNED** | 01-May-2024 |

| | |
|---|---|
| **GENERAL COMMENTS** | Thank you for the opportunity to review this paper. The authors have interesting data and analysis, however, I believe that major changes are needed before publication.<br><br>Abstract<br><br>The aim of this paper is more broad than stated. I suggest the impact of diabetes on patients at nutritional risk. The primary |

| | |
|---|---|
| | outcome was the effect of a nutritional intervention on these patients.<br>Methods: It is lacking the information that this study is a secondary analysis of previous study called EFFORT.<br>Result: I suggest that half of the result should show the comparison between diabetes and overall population and half between control and intervention.<br>Conclusion:<br>There was no significant difference between nutrition support and controls. However, when I read the conclusion I have a different impression.<br>As shown by the data, patients with diabetes seems to have more challenges for their care - worse glycemic control, more adverse effects, higher 180-day mortality.<br>Regarding the nutritional intervention the conclusion have to say no difference was found, but futher reaserch is needed to asses the best nutritional intervention for this population<br>Introduction<br>There is still room for research about nutritional support and that should be clearly stated. The last meta-analysis showed benefit, but with a lot of heterogeneity.<br>10.1001/jamanetworkopen.2019.15138<br>Again the aim of the study should be reviewed.<br><br>Methods<br>Please include the following information in the methodology section<br>Was there special nutritional support for patients with diabetes? (it is state in the limitation, but it should be here)<br>Was there a protocol for glycemic control in the in patients?<br>Please clarify:<br>Definition of diabetes was based on HbA1c (page 5 line 136). Did all patient in the study had it measured? If not was there an criteria for measurement?<br>Only the prespecified covariates were included in the logistic regression? The univariate analysis was not considered for the adjusment?<br><br>Result<br><br>After the brief description of the population, the next result should be the primary outcome.<br>NRS is a well established score no need for a paragraph and figure regarding its validity in this population. I phrase in the text and a supplemental figure is ok though.<br>Association of clinical outcomes and nutritional support - main finding.<br>The language is confusing. There was no statistical difference between groups. Please review it.<br>Figure 3: I would prefer a column for morning, noon and evening - with control and intervention side by side. Also, an overall mean of glucose values would be interesting. The p value of this analysis should be either on the legend or in figure.<br>Data regarding HbA1c is in the discussion but I could not find it.<br>There are index to assess the stress hyperglycemia (look at this paper: )<br><br>Discussion<br>Again language is leading to misinterpretation of the findings. There was no statistical benefit for nutritional intervention among patients with diabetes. |

| | |
|---|---|
| | A brief discussion about HbA1c from admission and previous glycemic control should be included (10.1038/s41598-019-55080-3; 10.1097/CCM.0b013e3181feb5ea)<br>The lack of protocol for the treatment of hyperglycemia should be included as a limitation, even though the best strategy is not yet defined (10.1159/000481355)<br><br>Conclusion<br>The conclusion should be revised to align more closely with the study findings. |

**VERSION 1 – AUTHOR RESPONSE**

Reviewer: 1
Dr. Eric Boersma, Thoraxcenter, Erasmus MC
Comments to the Author:
In the EFFORT randomised trial, Lancet 2019, the authors studied the effect of nutritional support in medical inpatients at nutritional risk. Nutritional support appeared associated with a reduced incidence of any ‘adverse clinical outcome’ at 30 days (primary endpoint) as compared with ‘standard hospital nutrition’. The adjusted odds ratio (OR) was 0·79 (95% CI 0·64-0·97). Also all-cause death was significantly reduced, with OR 0.65 (95% CI 0.47-0.91). The effect of the intervention on the primary endpoint in subjects with diabetes was reported in the main results paper (doi 10.1016/S0140-6736(18)32776-4), but the effect on mortality was not reported. The current paper presents a further analysis of the EFFORT trial, focussing on modifications of the effect of nutritional support on mortality between patients with versus without diabetes. There appeared no evidence of effect modification.

From methodological/statistical point of view I have the following questions and remarks:

1. Using the EFFORT data, the authors studied a) the association between diabetes and clinical outcomes, and b) the effect of randomisation to nutritional support (versus) in patients with diabetes. These are separate research questions, which need to be clearly distinguished. Also, the statistical methods to address these questions differ, e.g. with respect to the adjustment of potential confounders (and the selection of these confounders). The (current description of the) applied statistical methods fails to make this distinction and needs to be modified.
Reply: thank you – we agree and have now changed the text in abstract and methods to make this important distinction. We now define that our main objective is to look at the effect of nutrition on outcomes in the diabetic population and secondary, we also look at prognostic implications.

2. In view of the research goal, the specific attention (in the Results section) for the relation between nutritional severity risk and outcome is not clear (and is not addressed as such in the Methods section). It is also not clear why this analysis is limited to patients with diabetes only.
Reply: thank you – we focused on diabetes patients for this analysis because we have previously reported the prognostic implications of NRS and outcome in the overall population. We now updated the method section as suggested.

3. The authors state (Abstract and Methods) that the primary endpoint of EFFORT was 30-day all-cause mortality. However, the main results paper (see above) states that the primary endpoint was a composite of any ‘adverse clinical outcome(s)’, defined as ‘all-cause mortality, admission to intensive care, non-elective hospital readmission, major complications, and decline in functional status at 30 days’. The findings with regard to mortality as a single endpoint should be interpreted in that context.
Reply: thank you – this important point has now been changed in the methods section, which now reads: “The primary endpoint for this analysis was all-cause mortality within 30 days, which

corresponds to the principal secondary endpoint of the initial EFFORT analysis. [22] Secondary endpoints included adverse clinical outcomes such as admission to an intensive care unit (ICU), non-elective readmission after discharge, length of stay or decline in functional status measured by a decrease of at least 10% as measured by the Barthel index (primary endpoint of the initial trial).”

4. The authors use a mixture of Cox regression for ‘time-to-event analyses’ (hazard ratios [HR] are reported) and logistic regression for ‘binary outcomes’ (ORs are reported). However, the ‘binary outcomes’ occur during follow-up. Based on what criteria did the authors decide to use Cox versus logistic?. Note that the main results paper presents treatment effects in terms of odds ratios.
Reply: thank you – this is indeed an important point and needs revision – we now use hazard ratios for the analysis of mortality over 30 days and 180 days because we also present time to event analysis graphically with Kaplan Meier plots. All other outcomes are analysed with logistic regression analysis and reporting of odds ratios.

5. (How) did the authors evaluate the proportional hazard (PH) assumption with respect to the Cox model? And was the PH assumption satisfied?
Reply: thank you – yes, we indeed evaluated the proportional hazard (PH) assumption in the original trial and for this analysis and found that the assumption was satisfied.

6. ‘Continuous variables are expressed as mean ± standard deviation (SD) …’ (How) did the authors evaluate normality?
Reply: thank you – we used qnorm plots to visually check for normality and found normal distribution for the variables used for this analysis. The same approach was done for the initial trial reporting.

7. Regarding the effect of the intervention, the authors report HRs and ORs for patients with diabetes and for the overall cohort. They should also report estimates in patients without diabetes for comparison (see e.g. Table 2).
Reply: thank you – this information is now added to the table

8. The authors state (Abstract) that ‘… there was a 25%-reduction in the risk of mortality in patients with diabetes receiving nutritional support compared to controls (7% vs. 10%, adjusted HR 0.75, [95% CI 0.39 to 1.43]) …’. However, factually, a) this mortality reduction in the sub-population of patients with diabetes is not statistically significant, and b) there was no evidence of interaction. Hence, the best estimate of treatment effect is based on the entire sample of patients with and without diabetes.
Reply: thank you – we agree. In the overall sample there was a significant effect of nutritional support and lower mortality (which was published in the initial Lancet publication) and we found a similar trend in the subgroup of diabetic patients – with no evidence for subgroup effect. We thus conclude that – although the diabetic sample is too small and confidence intervals are large – diabetic patients have likely a similar mortality benefit compared to the overall population.

9. The authors state ‘In a first step, we analyzed the association between diabetes and clinical outcomes using unadjusted and adjusted regression models within the overall EFFORT patient cohort (n=2028).’ Was randomly allocated treatment included as a determinant of outcome in these models?
Reply: thank you – we did not included random allocation to the multivariate model. However, we did not see that the overall prognostic information (OR and HR) were different when adding this parameter to the model.

Reviewer: 2
Dr. Marina Verçoza Viana, Hospital de Clínicas de Porto Alegre
Comments to the Author:
Thank you for the opportunity to review this paper. The authors have interesting data and analysis,

however, I believe that major changes are needed before publication.

Abstract

The aim of this paper is more broad than stated. I suggest the impact of diabetes on patients at nutritional risk. The primary outcome was the effect of a nutritional intervention on these patients.
Reply: thank you – this information has now been updated and changed as suggested

Methods: It is lacking the information that this study is a secondary analysis of previous study called EFFORT.
Reply: thank you – this information has now been updated and changed as suggested

Result: I suggest that half of the result should show the comparison between diabetes and overall population and half between control and intervention.
Reply: thank you – we changed the reporting now as suggested

Conclusion:
There was no significant difference between nutrition support and controls. However, when I read the conclusion I have a different impression.
As shown by the data, patients with diabetes seems to have more challenges for their care - worse glycemic control, more adverse effects, higher 180-day mortality.
Regarding the nutritional intervention the conclusion have to say no difference was found, but futher reaserch is needed to asses the best nutritional intervention for this population
Reply: thank you – in the overall sample there was a significant effect of nutritional support and lower mortality and a similar trend in the subgroup of diabetic patients – with no evidence for subgroup effect. We thus conclude that – although the diabetic sample is too small and confidence intervals are large – diabetic patients have likely a similar mortality benefit compared to the overall population.
We agree that the effects are not significant in the diabetes subsample and we now rephrased to be more conservative in this regard.

Introduction
There is still room for research about nutritional support and that should be clearly stated. The last meta-analysis showed benefit, but with a lot of heterogeneity.
10.1001/jamanetworkopen.2019.15138
Reply: thank you – the introduction now reads: "Recently, several trials have found that nutritional support is beneficial for polymorbid medical patients, resulting in reduced risk for mortality and other complications, as well as improved functionality and quality of life with, however, important heterogeneity among trials."

Again the aim of the study should be reviewed.
Reply: thank you – we changed the aim to "Herein, to investigate the effect of nutritional support on mortality in hospitalized patients with diabetes and nutritional risk, we conducted a pre-planned secondary analysis in patients with diabetes participating in the Effect of early nutritional support on Frailty, Functional Outcomes, and Recovery of malnourished medical inpatients Trial (EFFORT).".

Methods
Please include the following information in the methodology section
Was there special nutritional support for patients with diabetes? (it is state in the limitation, but it should be here)
Reply: thank you – there was no specific protocols regarding nutritional support for patients with diabetes. This is now clarified in the text.

Was there a protocol for glycemic control in the in patients?
Reply: there was no specific protocols regarding nutritional support for patients with diabetes. This information was now added to the methods.

Please clarify:
Definition of diabetes was based on HbA1c (page 5 line 136). Did all patient in the study had it measured? If not was there an criteria for measurement?
Reply: within the initial trial, information on diabetes and routine HbA1C levels was collected in all patients. Measurement of HbA1C levels was not mandatory in all patients but based on the discretion of the treating physicians. For this analysis, we included patients with a previous diagnosis of diabetes and those that met the diagnosis of diabetes based on HbA1c value ≥6.5%.

Only the prespecified covariates were included in the logistic regression? The univariate analysis was not considered for the adjustment?
Reply: yes – this is correct – covariates were predefined based on the initial EFFORT protocol and analysis.

Result

After the brief description of the population, the next result should be the primary outcome.
Reply: thank you – we now changed the reporting of results

NRS is a well established score no need for a paragraph and figure regarding its validity in this population. I phrase in the text and a supplemental figure is ok though.
Reply: thank you – we now have included the illustration in the appendix and just mentioned this finding in the results

Association of clinical outcomes and nutritional support - main finding.
The language is confusing. There was no statistical difference between groups. Please review it.
Reply: thank you – this is now rephrased as suggested
Figure 3: I would prefer a column for morning, noon and evening - with control and intervention side by side. Also, an overall mean of glucose values would be interesting. The p value of this analysis should be either on the legend or in figure.
Data regarding HbA1c is in the discussion but I could not find it. There are index to assess the stress hyperglycemia (look at this paper: )
Reply: thank you – we changed it as suggested and included a new figure (Figure 3). Figure 3 now shows a column for morning, noon and evening with control and intervention side by side for the overall mean including the p-value of Wilcoxon-Mann-Whitney test. For the daily means we prefer to keep the actual figure (Figure 2). Detailed values for daily means can be found in the supplementary table 5.

Discussion
Again language is leading to misinterpretation of the findings. There was no statistical benefit for nutritional intervention among patients with diabetes.
Reply: thank you – we rephrased this section

A brief discussion about HbA1c from admission and previous glycemic control should be included (10.1038/s41598-019-55080-3; 10.1097/CCM.0b013e3181feb5ea)
Reply: thank you – changed as suggested

The lack of protocol for the treatment of hyperglycemia should be included as a limitation, even though the best strategy is not yet defined (10.1159/000481355)

Reply: thank you – this limitation was now added

Conclusion
The conclusion should be revised to align more closely with the study findings.
Reply: thank you – changed as suggested

## VERSION 2 – REVIEW

| | |
|---|---|
| **REVIEWER NAME** | ***Boersma, Eric*** |
| **REVIEWER AFFILIATION** | Thoraxcenter, Erasmus MC, Dept. of Cardiology |
| **REVIEWER CONFLICT OF INTEREST** | None |
| **DATE REVIEW RETURNED** | 24-Jun-2024 |

| | |
|---|---|
| **GENERAL COMMENTS** | I appreciate that the authors addressed my questions. I have no further remarks. |

| | |
|---|---|
| **REVIEWER NAME** | ***Viana, Marina Verçoza*** |
| **REVIEWER AFFILIATION** | Hospital de Clínicas de Porto Alegre |
| **REVIEWER CONFLICT OF INTEREST** | None |
| **DATE REVIEW RETURNED** | 09-Jun-2024 |

| | |
|---|---|
| **GENERAL COMMENTS** | Thank you to the authors for their efforts in improving this important paper. However, I still believe that the conclusions extend beyond the data presented. While the findings in this subpopulation align with the overall study, this paper specifically focuses on patients with diabetes, and no significant differences were found. The lack of statistical power should be addressed in the discussion. Therefore, I do not agree with the conclusions drawn by the authors, since it should focus on the analysis and data from this subpopulation. |

## VERSION 2 – AUTHOR RESPONSE

• PI Dr. Marina Verçoza Viana, Hospital de Clínicas de Porto Alegre Comments to the Author: Thank you to the authors for their efforts in improving this important paper. However, I still believe that the conclusions extend beyond the data presented. While the findings in this subpopulation align with the overall study, this paper specifically focuses on patients with diabetes, and no significant differences were found. The lack of statistical power should be addressed in the discussion. Therefore, I do not agree with the conclusions drawn by the authors, since it should focus on the analysis and data from this subpopulation. Reply: thank you for your response. We agree an have now reworded the conclusion in abstract and main text as follows: "Conclusion: Our secondary analysis revealed no significant impact on mortality from individualized nutritional support in patients with diabetes, nor did it indicate any diabetes-associated side effects. To determine if the positive trends observed in this study are statistically significant, further large-scale trials are required." We hope that this wording is now acceptable and better represents the results of our study. thank you
